# Silent brain ischemia within the TAXINOMISIS framework: association with clinical and advanced ultrasound metrics

**DOI:** 10.3389/fneur.2024.1424362

**Published:** 2024-10-11

**Authors:** Vassiliki Kigka, Alessandro Carrozzi, Laura Ludovica Gramegna, Panagiotis K. Siogkas, Vassiliki Potsika, Vassilis Tsakanikas, Michael Kallmayer, Victor Obach, Vincente Riambau, Giovanni Spinella, Giovanni Pratesi, Luigi Cirillo, David Neil Manners, Rodolfo Pini, Gianluca Faggioli, Gert J. de Borst, George Galyfos, Frangiska Sigala, Perica Mutavdzic, Marija Jovanovic, Igor Koncar, Dimitros I. Fotiadis, on behalf of the TAXINOMISIS Silent Ischemia Working Group

**Affiliations:** ^1^Unit of Medical Technology and Intelligent Information Systems, Department of Materials Science and Engineering, University of Ioannina, Ioannina, Greece; ^2^Department of Biomedical Research-FORTH, Institute of Molecular Biology and Biotechnology, University Campus of Ioannina, Ioannina, Greece; ^3^Department of Medical and Surgical Sciences (DIMEC), University of Bologna, Bologna, Italy; ^4^Vall d'Hebron Research Institute, Vall d'Hebron Barcelona Hospital Campus, Barcelona, Spain; ^5^Neuroradiology Unit, Radiology Department, Hospital del Mar, Barcelona, Spain; ^6^Department of Vascular and Endovascular Surgery, School of Medicine & Klinikum rechts der Isar, Technical University of Munich, Munich, Germany; ^7^Fundacio per la Recerca Clinic Barcelona-IDIBAPS (FRCB-IDIBAPS), Barcelona, Spain; ^8^Vascular and Endovascular Surgery Clinic, Ospedale Policlinico San Martino, Genoa, Italy; ^9^Department of Integrated Surgical and Diagnostic Sciences (DISC), University of Genova, Genoa, Italy; ^10^Functional and Molecular Neuroimaging Unit, IRCCS Istituto delle Scienze Neurologiche di Bologna, Bologna, Italy; ^11^Department of Biomedical and Neuromotor Sciences (DIBINEM), University of Bologna, Bologna, Italy; ^12^Department for Life Quality Studies (QUVI), University of Bologna, Bologna, Italy; ^13^Vascular Surgery, IRCCS Policlinico Sant’Orsola-Malpighi, Bologna, Italy; ^14^Department of Vascular Surgery, University Medical Center Utrecht, Utrecht, Netherlands; ^15^Vascular Unit, First Department of Propaedeutic Surgery, National and Kapodistrian University of Athens, Athens, Greece; ^16^Faculty of Medicine, University of Belgrade, Belgrade, Serbia; ^17^Clinic for Vascular and Endovascular Surgery, University Clinical Centre of Serbia, Belgrade, Serbia

**Keywords:** silent brain infarcts (SBIs), carotid artery stenosis (CAS), risk factors, magnetic resonance imaging (MRI), carotid ultrasound, computational fluid dynamics (CFD)

## Abstract

**Introduction:**

The relationship between carotid artery stenosis (CAS) and ipsilateral silent brain ischemia (SBI) remains unclear, with uncertain therapeutic implications. The present study, part of the TAXINOMISIS project (nr. 755,320), aimed to investigate SBIs in patients with asymptomatic CAS, correlating them with clinical, carotid ultrasonographic data, and CFD analyses.

**Methods:**

The TAXINOMISIS clinical trial study (nr. NCT03495830) involved six vascular surgery centers across Europe, enrolling patients with asymptomatic and symptomatic CAS ranging from 50 to 99%. Patients underwent carotid ultrasound and magnetic resonance imaging (MRI), including brain diffusion-weighted, T2-weighted/FLAIR, and T1-weighted sequences. Brain MRI scans were analyzed for the presence of SBI according to established definitions. Ultrasound assessments included Doppler and CFD analysis. Only asymptomatic patients were included in this substudy.

**Results:**

Among 195 asymptomatic patients, the mean stenosis (NASCET) was 64.1%. Of these, a total of 33 patients (16.9%) had at least one SBI detected on a brain MRI scan. Specifically, 19 out of 33 patients (57.6%) had cortical infarcts, 4 out of 33 patients (12.1%) had ipsilateral lacunar infarcts, 6 out of 33 patients had (18.2%) subcortical infarcts, 1 out of 33 patients (3.0%) had both cortical and lacunar infarcts, and 3 out of 33 patients (9.1%) both cortical and subcortical infarcts. Patients with SBIs exhibited significantly higher risk factors, including a higher body mass index (28.52 ± 9.38 vs. 26.39 ± 3.35, *p* = 0.02), diastolic blood pressure (80.87 ± 15.73 mmHg vs. 80.06 ± 8.49 mmHg, p = 0.02), creatinine levels (93.66 ± 34.61 μmol/L vs. 84.69 ± 23.67 μmol/L, p = 0.02), and blood triglycerides (1.8 ± 1.06 mmol/L vs. 1.48 ± 0.78 mmol/L, *p* = 0.03). They also had a higher prevalence of cardiovascular interventions (29.6% vs. 13.8%, *p* = 0.04), greater usage of third/fourth-line antihypertensive treatment (50%vs16%, p = 0.03), and anticoagulant medications (60% vs. 16%, *p* = 0.01). Additionally, the number of contralateral cerebral infarcts was higher in patients with SBIs (35.5% vs. 13.4%, *p* < 0.01). Moreover, carotid ultrasound revealed higher Saint Mary’s ratios (15.33 ± 12.45 vs. 12.96 ± 7.99, *p* = 0.02), and CFD analysis demonstrated larger areas of low wall shear stress (WSS) (0.0004 ± 0.0004 m^2^ vs. 0.0002 ± 0.0002 m^2^, p < 0.01).

**Conclusion:**

The TAXINOMISIS clinical trial provides valuable insights into the prevalence and risk factors associated with SBIs in patients with moderate asymptomatic carotid stenosis. The findings suggest that specific hemodynamic and arterial wall characteristics may contribute to the development of silent brain infarcts.

## 1 Introduction

Carotid artery stenosis (CAS) is a primary cause of ischemic cerebrovascular events, responsible for approximately 150,000 deaths annually in Europe and approximately 130,000 in the United States due to stroke, contributing significantly to health consequences and long-term disability (1). The socioeconomic impact of these outcomes is substantial.

European Stroke Organization (ESO) guidelines recommend statin therapy as a standard treatment for CAS. However, the 2023 European Society for Vascular Surgery (ESVS) (2) guidelines suggest considering carotid endarterectomy (CEA) for asymptomatic CAS patients who show signs of silent brain ischemia (SBI) on imaging (3).

While the association between CAS and symptomatic stroke has been extensively studied, the relationship between CAS and clinically asymptomatic ischemic events, commonly defined as SBI, remains unclear.

SBIs typically occur without noticeable symptoms, posing a diagnostic challenge for general practitioners. These findings, often seen in brain MRI reports, are frequently considered incidental and of uncertain clinical significance, leading to minimal changes in patient management (4).

The prevalence of SBIs in patients with CAS is estimated to be approximately 10–20% (5). Although some studies have suggested a correlation between internal carotid artery intima-media thickness and stenosis with the occurrence of SBI (6), the exact mechanism by which atherothrombotic emboli lead to SBI remains unclear.

Recent advances in computational fluid dynamics (CFD) have improved our understanding of atherosclerosis, demonstrating that areas of low wall shear stress (WSS) are associated with increased accumulation of LDL particles within the arterial walls, which may contribute to the formation of atherosclerotic plaques (7).

The TAXINOMISIS project aims to classify carotid artery disease by leveraging current patient data and conducting prospective clinical trials. This involves characterizing plaque lesions, identifying risk factors, and analyzing disease phenotypes. The second phase includes a prospective clinical study to collect data on carotid stenosis and brain tissue.

The current study aims to describe the presence of SBIs in the TAXINOMISIS cohort and to correlate these findings with clinical and ultrasonographic characteristics, including advanced CFD analysis of the carotid plaque.

## 2 Materials and methods

Asymptomatic patients from the TAXINOMISIS core study cohort were included in this substudy. The TAXINOMISIS study was a prospective, observational, multicenter trial that enrolled individuals with moderate to severe extracranial CAS ranging from 50 to 99%. The study included both asymptomatic and symptomatic patients and was conducted at six European vascular centers: Athens-NKUA, Barcelona-FCRB, Belgrade-UBEO, Genoa-USMI, Munich-TUM, and Utrecht-UMC. The enrollment period extended from March 30, 2018, to December 31, 2019, with a total of 345 patients. The trial is registered on ClinicalTrials.gov under the identifier NCT03495830, and a detailed protocol has been published by Timmerman et al. (8).

### 2.1 Demographic and clinical data collection

Demographic, clinical, and blood analysis data were collected from all patients. The treating physician determined whether the patient was symptomatic or asymptomatic based on the presence of clinical signs or a history of previous stroke or transient ischemic attack (TIA).

### 2.2 Ultrasonographic examination

All patients underwent an ultrasonographic examination, which included the assessment of carotid stenosis (using NASCET criteria), plaque morphology, and peak systolic velocity (PSV) in both the internal carotid artery (ICA) and common carotid artery (CCA). Additionally, St. Mary’s ratio—calculated as the ratio of PSV in the ICA to the end-diastolic velocity (EDV) in the CCA—was measured.

### 2.3 MRI examination

**Figure 1:**
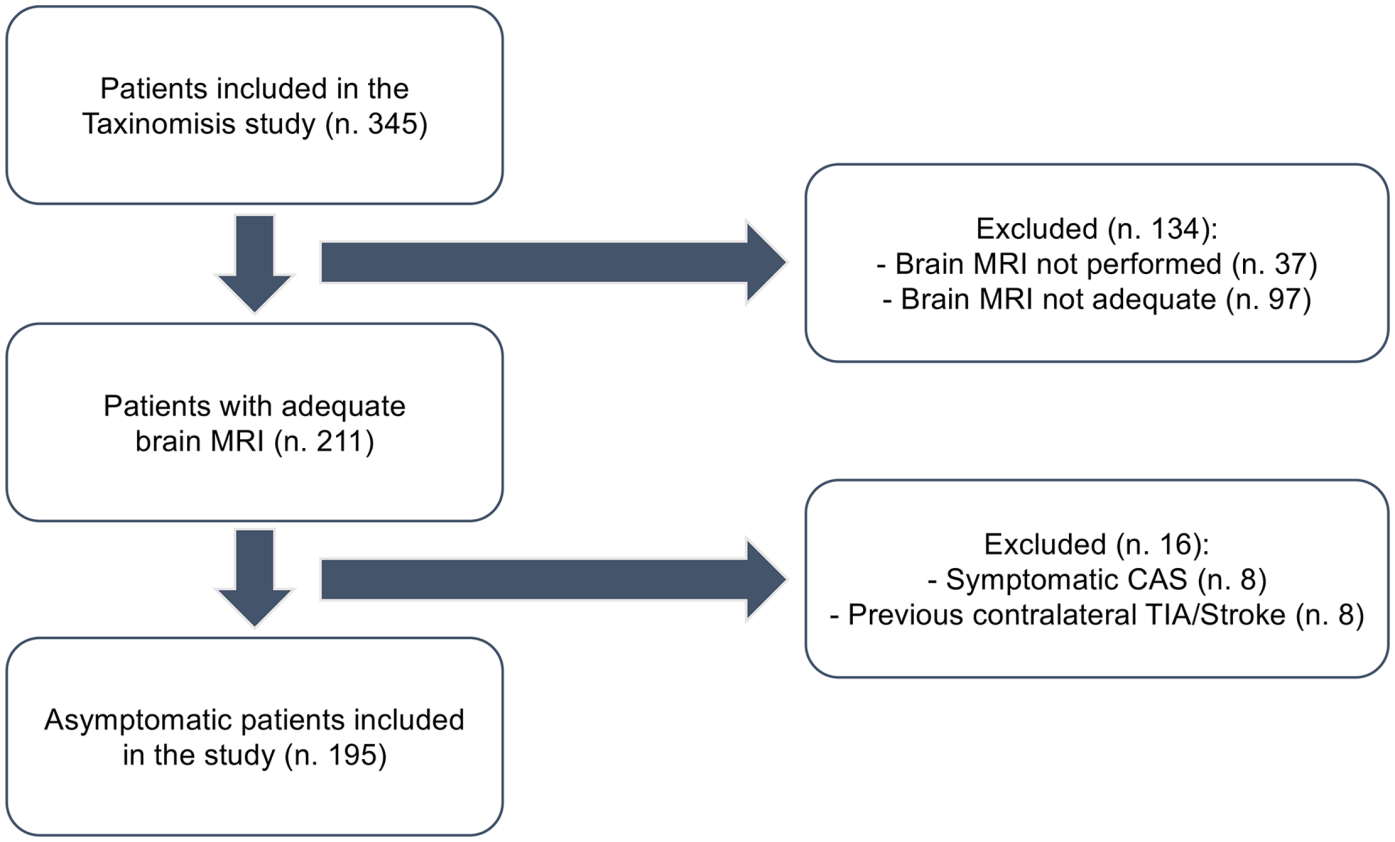
Flowchart describing the inclusion process of patients in the study. Adequate: at least T1 and T2/FLAIR images performed.

All patients underwent a baseline MRI examination on a 3 T scanner. The scan protocol included at least one axial diffusion-weighted imaging (DWI) scan and a time-of-flight (ToF) image. In contrast, an axial fluid-attenuated inversion recovery (FLAIR) T2-weighted image and a T1-weighted image were optional. Data in Digital Imaging and Communications in Medicine (DICOM) format from each center were anonymized, collected, and sent to an external core lab at the Neuroimaging Laboratory at the University of Bologna (IRCCS Istituto delle Scienze Neurologiche di Bologna), which conducted the analysis. Images deemed non-diagnostic (i.e., lacking sequences, noisy, or containing motion/distortion artifacts) were excluded. A flow chart regarding the inclusion process is displayed in Figure 1.

Two expert neuroradiologists, with 2 and 15 years of experience, respectively, reviewed the images and assessed the presence of ipsilateral brain infarcts in the anterior circulation vascular territory by consensus; they were also blinded to the clinical data. Infarcts were divided into three categories based on their location: cortical, subcortical, and lacunar, according to the previous definition of SBI (5, 9). A lesion was considered cortical if it presented as a FLAIR/T2-weighted hyperintensity affecting the cerebral cortex, regardless of its shape or size.

**Figure 2:**
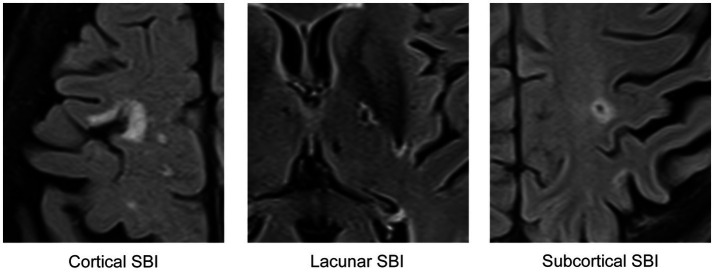
FLAIR/T2-weighted images displaying representative examples of the three distinct categories of brain infarcts utilized in this study. Each depicted infarct is chronic and was identified in asymptomatic patients, classifying them as SBIs.

Subcortical infarcts were defined as round or ovoid lesions that were hyperintense on T2-weighted/FLAIR images with a central fluid-filled cavity (i.e., with signal intensity similar to cerebrospinal fluid (CSF), measuring between 3 mm and 15 mm in diameter, located in the vascular territory of superficial perforating arteries). Lacunar infarcts shared the same characteristics as subcortical infarcts but were located in the territory of the deep perforating arteries. Figure 2 illustrates a representative example of each infarct type. Infarcts were classified as acute/subacute if they exhibited significant diffusion restriction on a DWI scan (i.e., a hyperintense signal on a DWI scan corresponding to a low signal on the ADC map) and as chronic if no changes were observed on DWI scans or ADC maps.

### 2.4 CFD analysis

A 3D reconstruction of the arterial lumen was conducted using a custom-developed algorithm to calculate the necessary hemodynamic parameters. Briefly, the ToF sequence from the MRI scan was used to generate a 3D volume of the arterial lumen (10). Subsequently, finite element method (FEM) analysis was conducted using ANSYS 16.2^®^. The Navier–Stokes and continuity equations were used to model the blood flow.

Blood was modeled as a Newtonian fluid with a density of 1,050 kg/m^3^ and a dynamic viscosity of 0.0035 Pa·s. Transient blood flow simulations were conducted for all cases. More specifically, the 3D models were discretized into tetrahedral finite elements with a maximum face size of 0.16 mm, which was determined using mesh sensitivity analysis. Ultrasound scans provided flow velocity data for the CCA and external carotid artery (ECA), which were used to generate mass flow rate profiles, serving as boundary conditions for the FEM blood flow simulations.

A zero-pressure boundary condition was used at the ICA outlet, with the arterial wall assumed to be rigid and impermeable. Patient-specific mass flow rate values were used for a complete cardiac cycle, from which key hemodynamic parameters were derived. These included time-averaged wall shear stress (TAWSS), oscillatory shear index (OSI), pressure ratios (PICA/PCCA and PECA/PCCA), along with the areas of low TAWSS and high OSI, both normalized by the total vessel area. These parameters were calculated for all 195 arterial models (11).

### 2.5 Statistical analysis

Statistical analysis was conducted using IBM SPSS version 23.0 (Statistical Package for the Social Sciences, version 23.0 for Windows, 2015). The normal distribution of continuous variables was assessed using the Kolmogorov–Smirnov and Shapiro–Wilk tests. Descriptive statistics were presented as mean (SD) for continuous variables and number (%) for categorical variables.

All analyses were conducted at the patient level, incorporating echographic data from the stenosed carotid artery for which the patient was included, data from the ipsilateral cerebral hemisphere, and clinical information. Clinical histories were meticulously reviewed to ascertain the presence of symptoms related to each affected carotid artery. Patients with neurological symptoms from both carotid arteries were excluded. If a patient had a contralateral silent brain infarct, this parameter was recorded as “contralateral brain infarct.” For asymptomatic patients, we compared echographic characteristics and clinical data between those with at least one sign of silent brain infarct and those without.

Continuous variables between Class 1 (presence of SBIs) and control Class 0 (absence of SBIs) were analyzed using an independent-sample t-test, while categorical data were compared using the Chi-square test and odds ratio (OR) analysis. *p*-values of < 0.05 were considered to be statistically significant. Additionally, multiple regression analysis was also conducted to verify the independence of the utilized features.

The above-mentioned analysis was repeated for each type of brain infarct (cortical, small subcortical, and lacunar).

## 3 Results

The descriptions of both the continuous and discrete utilized features are shown in Table 1.

**Table 1:** Summary of patients’ demographics, clinical information, and findings from the carotid ultrasound examinations.

Overall cohort (N = 195)			
ICA stenosis, mean (SD)	64.13 (13.6)	Sex (male), n (%)	121 (62.05%)
PSV, mean (SD)	177.57 (97.51)	Alcohol abuse, n (%)	19 (9.74%)
PSV ICA / PSV CCA, mean (SD)	3.81 (5.79)	Diabetes, n (%)	57 (29.23%)
St Mary’s ratio, mean (SD)	13.43 (8.98)	Hypertension, n (%)	166 (85.13%)
P_ICA_/P_CCA_, mean (SD)	0.93 (0.15)	Coronary disease, n (%)	45 (23.08%)
P_ECA_/P_CCA_, mean (SD)	0.9 (0.28)	Previous MI, n (%)	17 (8.72%)
Vessel average TAWSS, mean (SD)	10.84 (15.86)	Previous CABG/PCI, n (%)	27 (13.85%)
Area of low TAWSS (m^2^), mean (SD)	0.0002 (0.0002)	Atherosclerosis of aortoiliac segment or femoropoplitealcrural, n (%)	48 (24.62%)
Area of low TAWSS/Total vessel area (%), mean (SD)	25.23 (18.73)	Alpha-blockers (no), n (%)	6 (3.08%)
Area of high OSI/Total vessel area (%), mean (SD)	26.08 (18.13)	Beta-blockers, n (%)	105 (53.85%)
Height, mean (SD)	168.63 (15.69)	Diuretics, n (%)	58 (29.74%)
Weight, mean (SD)	76.8 (14.56)	Calcium channel blockers, n (%)	48 (24.62%)
BMI, mean (SD)	26.75 (4.99)	ACE inhibitors, n (%)	87 (44.62%)
SBP, mean (SD)	131.06 (18.21)	Angiotensin receptor antagonist, n (%)	38 (19.49%)
DBP, mean (SD)	80.17 (10.01)	Other antihypertensive drugs, n (%)	15 (7.69%)
Pulse rate, mean (SD)	70.67 (7.35)	Coronary drugs, n (%)	10 (5.13%)
Age, mean (SD)	70.09 (7.6)	Anticoagulants, n (%)	5 (2.56%)
Hb, mean (SD)	11.99 (2.86)	Acetylsalicylic acid, n (%)	171 (87.69%)
Hct, mean (SD)	40.58 (4.68)	Clopidogrel, n (%)	27 (13.85%)
Creatinine, mean (SD)	86.07 (25.73)	Statin, n (%)	174 (89.23%)
Cholesterol, mean (SD)	4.59 (1.12)	Lipid-lowering drugs, n (%)	22 (11.28%)
LDL, mean (SD)	2.54 (1.002)	Contralateral, n (%)	31 (15.9%)
HDL, mean (SD)	1.5 (0.56)	Ever operated on contralateral carotid, n (%)	32 (16.41%)
Triglycerides, mean (SD)	1.53 (0.83)	Cortical or small subcortical or lacunar, n (%)	33 (16.92%)
Glucose, mean (SD)	6.41 (2.32)		
CRP, mean (SD)	3.33 (3.69)		
HbA1c, mean (SD)	6.04 (0.9)		

### 3.1 Baseline study

A total of 195 asymptomatic patients were included in the study. Of these, 33 patients (16.9%) had 36 asymptomatic ipsilateral brain infarcts. Specifically, 19 of the 33 patients (57.6%) had cortical infarcts, 4 patients (12.1%) had ipsilateral lacunar infarcts, 6 patients (18.2%) had subcortical infarcts, 1 patient (3.0%) had both a cortical and a lacunar infarct, and 3 patients (9.1%) had both cortical and subcortical infarcts.

A detailed breakdown of the MRI findings for each clinical center can be found in Supplementary material, Part 1.

### 3.2 Association between SBIs and clinical and US data

The results of the overall cohort (patient-level analysis) are reported in Tables 2, 3. Geographical, computational and clinical factors associated with the presence of SBIs include higher mean values of St Mary’s ratio (15.33 ± 12.45 vs. 12.96 ± 7.99, *p* = 0.02), area of low TAWSS (0.0004 ± 0.0004 m^2^ vs. 0.0002 ± 0.0002 m^2^, *p* < 0.01), BMI (28.52 ± 9.38 vs. 26.39 ± 3.35, *p* = 0.02), diastolic blood pressure (80.87 ± 15.73 mmHg vs. 80.06 ± 8.49 mmHg, *p* = 0.02), creatinine (93.66 ± 34.61 μmol/L vs. 84.69 ± 23.67 μmol/L, *p* = 0.02), and blood triglycerides (1.8 ± 1.06 mmol/L vs. 1.48 ± 0.78 mmol/L, *p* = 0.03).

**Table 2:** Subjects’ continuous characteristics in the absence or presence of SBIs and odds ratio analysis for the association of cardiovascular-related risk factors and the presence of brain lesions.

Continuous features	Absence of SBIs (N = 162)	Presence of SBIs (N = 33)	*p* value
ICA stenosis	63.59 ± 13.81	65.86 ± 12.17	0.767
PSV	172.06 ± 93.12	190.57 ± 98.02	0.32
PSV ICA / PSV CCA	3.85 ± 6.34	3.76 ± 2.12	0.811
St Mary’s ratio	12.96 ± 7.99	15.33 ± 12.45	**0.016**
Vessel average TAWSS (Pa)	11.83 ± 17.49	6.86 ± 3.35	0.269
Area of low TAWSS (m^2^)	0.0002 ± 0.0002	0.0004 ± 0.0004	**0.002**
Area of low TAWSS/Total vessel area (%)	23.001 ± 17.099	35.17 ± 23.45	0.404
Area of high OSI/Total vessel area (%)	25.93 ± 19.84	26.95 ± 8.87	0.255
Weight	75.75 ± 11.8	81.8 ± 18.52	0.409
BMI	26.39 ± 3.35	28.52 ± 9.38	**0.022**
Systolic BP	130.97 ± 16.48	130.73 ± 25.21	0.106
Diastolic BP	80.06 ± 8.49	80.87 ± 15.73	**0.025**
Pulse rate	70.67 ± 7.52	70.36 ± 6.48	0.702
Age	69.89 ± 7.64	71.48 ± 7.05	0.578
Creatinine	84.69 ± 23.67	93.66 ± 34.61	**0.022**
Cholesterol	4.67 ± 1.104	4.27 ± 1.15	0.872
LDL	2.6 ± 1.02	2.3 ± 0.85	0.364
HDL	1.52 ± 0.56	1.39 ± 0.58	0.860
Triglycerides	1.48 ± 0.78	1.8 ± 1.06	**0.031**
Glucose	6.42 ± 2.28	6.39 ± 2.55	0.433
CRP	3.49 ± 3.9	2.68 ± 2.49	0.381
(GHbA1c)	6.02 ± 0.84	6.17 ± 1.24	0.283

Bold font indicates statistical significance.

**Table 3:** Subjects’ categorical characteristics in the presence of SBIs and odds ratio analysis for the association of cardiovascular-related risk factors and the presence of brain lesions.

Categorical features	N. of SBIs (%)	Odds ratio—CI	*p* value
Men	25 (21%)	0.456 (0.194–1.073)	0.067
Women	8 (10.8%)		
Alcohol abuse	3(15.8%)	0.869 (0.238–3.171)	0.831
Diabetes	11(19.3%)	1.239 (0.556–2.76)	0.599
Hypertension	28 (17.1%)	0.947 (0.332–2.704)	0.919
Coronary Disease	10 (22.2%)	1.729 (0.741–4.033)	0.202
Previous MI	4 (23.5%)	1.669 (0.505–5.519)	0.397
Previous Cabg/pci	8 (29.6%)	2.622 (1.023–6.717)	**0.039**
Aortoiliac atherosclerosis /femoropoplitealcrural	6 (12.5%)	0.611 (0.234–1.597)	0.312
Aortic aneurysm	2 (25%)	1.714 (0.329–8.933)	0.518
Alpha-Blockers	3 (50%)	5.233 (1.008–27.175)	0.03
Beta-Blockers	20 (19.4%)	1.39 (0.647–2.987)	0.397
Diuretics	8 (13.8%)	0.691(0.291–1.64)	0.4
Ca channel blockers	8 (17%)	0.976 (0.407–2.341)	0.957
ACE inhibitors	11 (12.6%)	0.526 (0.239–1.158)	0.107
Angiotensin II receptor antagonist	8 (21.6%)	1.423 (0.583–3.474)	0.436
Other antihypertensive drugs	2 (14.3%)	0.785 (0.167–3.685)	0.758
Coronary drugs	3 (30%)	2.157 (0.528–8.819)	0.274
Anticoagulants	3 (60%)	7.9 (1.266–49.311)	**0.01**
Acetylsalicylic acid	28 (16.5%)	0.631 (0.214–1.863)	0.401
Clopidogrel	6 (23.1%)	1.533 (0.563–4.173)	0.4
Statin	29 (16.9%)	0.76 (0.235–2.458)	0.646
LLD	5 (23.8%)	1.632 (0.551–4.831)	0.373
Contralateral	11 (35.5%)	3.55 (1.49–8.406)	**0.003**
Ever operated on contralateral carotid	4 (12.9%)	0.747 (0.24–2.322)	0.613

Bold font indicates statistical significance.

Moreover, positive risk factors for the presence of brain lesions were the previous CABG/PCI placement (OR: 2.622, CI: 1.023–6.717, *p*-value: 0.039), the use of alpha-blockers (OR: 5.233, CI: 1.008–27.175, *p*-value: 0.03), the use of anticoagulants (OR: 7.9, CI:1.266–49.311, *p*-value: 0.01), and the presence of contralateral brain lesions (OR: 3.55, CI: 1.49–8.406, *p*-value: 0.003).

**Figure 3:**
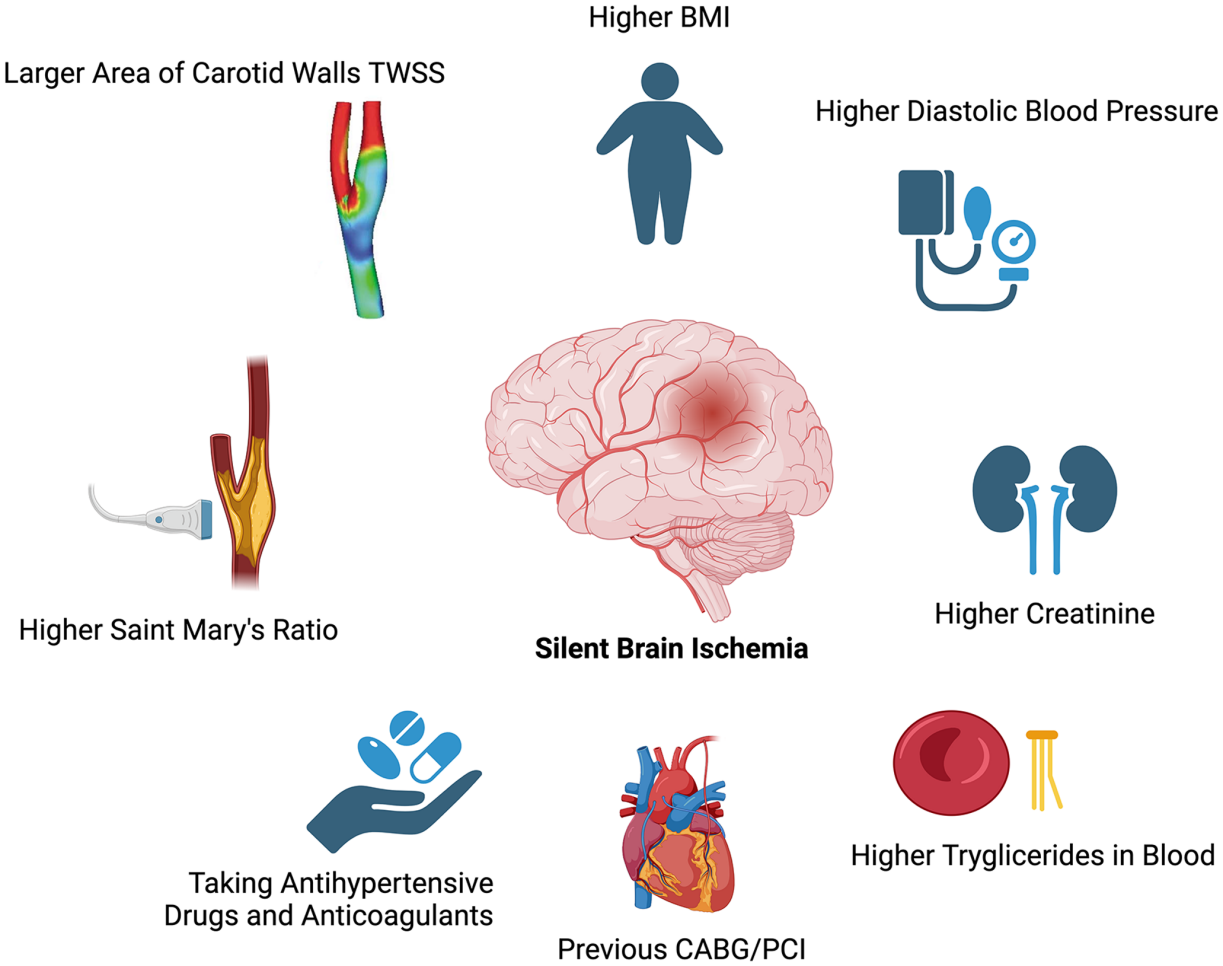
Association of patient-specific features with the presence of SBIs.

The study’s findings regarding the association of each patient’s features with the SBI presence are depicted in Tables 2, 3 and visually represented in Figure 3.

The respective results regarding the presence of cortical, small subcortical, and lacunar infarcts are shown in Supplementary Tables S1, S2.

## 4 Discussion

In our study, we found that SBIs were associated with a higher prevalence of cardiovascular risk factors. Additionally, the association with lower WSS provides further insight into the pathophysiology of the disease, suggesting that this type of ischemia may be linked to more advanced stages of CAS.

The prevalence of SBI in our cohort is in line with existing literature (5, 12), which reports rates ranging from 10 to 20% and approximately 17–33% in patients with CAS. We did not observe any difference in the degree of CAS between patients with and without signs of SBI, with values of 63.59 ± 13.81% and 65.86 ± 12.17%, respectively. This suggests that carotid stenosis alone, commonly used as a stratification criterion, may be insufficient for a comprehensive assessment of these patients.

Over the last few years, it has become increasingly evident that carotid stenosis severity alone is insufficient to predict carotid plaque instability, necessitating the exploration of alternative indicators for a more accurate prognostic evaluation of plaque morphology (13). In a study conducted by Kakkos et al. (14) involving 821 patients with asymptomatic CAS, those with SBIs experienced a twofold increase in stroke events over an 8-year follow-up compared to those without SBIs. Furthermore, there is an established association between asymptomatic CAS and cognitive impairment, which may be explained by the presence of SBIs resulting from atheroembolic phenomena (9).

This study demonstrated that patients with SBIs exhibited a significantly higher Saint Mary’s ratio compared to those without SBIs. This ratio is considered a robust indicator of carotid disease severity in Doppler ultrasound assessments (15), as it reflects the hemodynamic changes caused by plaque. It is calculated by dividing the peak systolic velocity (PSV) in the internal carotid artery (ICA), which increases with the degree of stenosis, by the end-diastolic velocity (EDV) in the distal CCA, which decreases as ICA resistance rises with advancing stenosis.

Typically, a value greater than 14 predicts stenosis exceeding 70%, and a ratio above 11 indicates stenosis greater than 60% (15). In our study, the mean value across the entire cohort was only under 14, but it increased to 15 among patients with silent ischemia, despite no significant increase in stenosis, suggesting that the rise in values was attributed to a reduction in the numerator (i.e., EDV in the distal CCA) resulting from decreased vessel compliance, which is primarily caused by atherosclerosis (16).

From a clinical point of view, our results suggest that SBIs could still serve as markers for identifying individuals at elevated cardiovascular risk among those with asymptomatic CAS.

Within our study cohort, patients with SBIs showed increased use of third- and fourth-line antihypertensive treatments, along with elevated diastolic blood pressure and triglyceride levels, suggesting the need for optimizing medical therapy and considering prophylactic carotid interventions. The European Stroke Organization (ESO) guidelines on covert SVD recommend addressing vascular risk factors upon SBI detection to prevent major cardiovascular events and stress the importance of lifestyle changes. They also propose statin therapy even without traditional indications (2). The 2023 European Society for Vascular Surgery (ESVS) guidelines on carotid management suggest considering CEA for patients with SBIs identified through CT or MRI, despite asymptomatic CAS and optimal medical therapy (3).

Moreover, building on recent insights from CFD into the mechanisms of atherosclerosis, particularly in identifying regions susceptible to plaque development, we conducted a novel investigation to examine distinct CFD analysis parameters in asymptomatic patients with CAS for the first time.

We found that patients with SBIs exhibit a significantly larger area characterized by low WSS in the carotid artery ipsilateral to the infarct, compared to those without SBIs. Recent studies suggest that low WSS is a critical factor in the initiation of atherosclerosis (17), primarily by stimulating an inflammatory response in endothelial cells and upregulating adhesion molecules and chemokines, which are critical to atherogenesis (18).

Furthermore, atherosclerotic regions exposed to low WSS, particularly those rich in lipids, tend to exhibit accelerated plaque progression (19) and increased plaque burden (20). Studies on animal models have shown that low WSS is commonly found in plaques with high-risk characteristics, such as a large lipid core and a thin fibrous cap of smooth muscle cells and collagen (21). Recent studies have suggested that low WSS promotes atherosclerosis by inducing the formation of neutrophil extracellular traps (NETs) through the Piezo1-HDAC2 pathway (22) and triggering endothelial cell pyroptosis via the IKKε/STAT1/NLRP3 pathway (23).

Additionally, an *in vivo* study involving 20 patients with non-occlusive coronary artery disease (CAD) demonstrated that low WSS regions within plaques were associated with a regression of fibrous and fibro-lipidic content and an expansion of the necrotic core, further progressing toward a high-risk phenotype (24). However, some evidence suggests that high-risk plaque features, particularly ulceration and intraplaque hemorrhage (IPH), are associated with areas of elevated WSS (25, 26).

Plaques frequently exhibit a distinctive spatial pattern, with high WSS regions located upstream and low WSS areas downstream (27). This pattern suggests a complex interaction between WSS and plaque vulnerability, where plaque development is likely shaped by a combination of hemodynamic and biological factors (17).

It is interesting, though unsurprisingly, that all observed SBIs, regardless of their type, exhibited characteristics consistent with chronic-phase ischemia, with no acute lesions found on DWI scans. This is likely due to the older age of our study cohort (average age approximately 70 years). Additionally, over the 3-year follow-up, only very few new SBIs were identified. Our findings align with the notion that SBIs can occur relatively early in the cardiovascular risk trajectory.

This is in line with the results of the PESA study, which examined 4,184 middle-aged healthy participants and found early signs of atherosclerosis: 36% of men and 25% of women aged 45–49 years had significant atherosclerotic plaques in the carotid arteries (28).

There is currently no consensus on the diagnostic criteria for SBIs. SBIs are usually defined on brain MRI as lesions with CSF-like signals: hypointense on T1-weighted and hyperintense on T2-weighted/FLAIR images, with a diameter of at least 3 mm, excluding dilated perivascular spaces and white matter hyperintensities (WMH) (5, 6, 12). This definition was used in the review conducted by the clinical neuroradiologists for this study.

Moreover, through this study, we further analyzed the types of brain infarcts in this population, distinguishing between cortical and subcortical infarcts (6) and lacunae within the territory of the vascularization of the proximal penetrating arteriole.

Regarding cortical SBIs, substantial evidence points to atheroembolic processes as the primary cause of cortical ischemia in individuals with CAS (4). Similarly, other data indicate that subcortical infarcts in the territory of distal perforating arteries are often associated with atherosclerosis and stenosis in the major brain-feeding arteries (29–31).

Lacunes within the territory of proximal penetrating arteriole vascularization are frequently correlated with other indicators of small vessel disease (32). However, they may also be associated with embolization from the carotid and have previously been recognized as signs of SBI (33).

Moreover, our results are in line with previous data (34) involving 347 patients with brain lesions classified as infarcts on MRI scans, both cortical and subcortical. Only 14% had corresponding clinical symptoms.

It is well known that the presence of SBIs is associated with greater mean carotid intima-media thickness (IMT) (5), as well as with stenosis (6) and high-risk plaques (5, 35). Whether carotid stenosis solely indicates an overall elevated cardiovascular risk or directly contributes, through plaque rupture and subsequent atheroembolism, to SBIs remains uncertain.

Our data appear to support both possibilities, as the presence of SBIs was associated with cardiovascular risk factors and hemodynamic features (such as the St. Mary’s ratio and wall shear stress) that suggest hemodynamic perturbations may support the formation of atheroembolism.

### 4.1 Limitations and future directions

This study has several limitations. A possible one is the lack of evaluation for microembolic signals via transcranial Doppler, followed by IPH detection using carotid MRI or computed tomography. Additionally, several brain MRIs had to be excluded due to the lack of FLAIR images, which were deemed necessary for visualizing small infarctions and SBIs.

The NASCET method, while commonly used, has certain limitations in assessing carotid stenosis. Specifically, it is well known that it may not fully capture the degree of stenosis in cases involving outward plaque remodeling (36, 37). Additionally, the NASCET method is known for high interobserver variability (38). Despite these drawbacks, it remains one of the most widely used methods in clinical practice, making it valuable for translating our findings into clinical reasoning.

A comprehensive risk assessment that incorporates these factors, along with SBI presence, might be essential for accurately defining patient risk profiles and determining optimal therapeutic strategies (3, 13). Further research on the subject is needed, including the use of emerging imaging modalities, such as PET-CT (39) and intravascular OCT (40), to better characterize patient risk and refine therapeutic management strategies.

## 5 Conclusion

The TAXINOMISIS clinical trial provides crucial insights into the prevalence and risk factors of ipsilateral silent brain infarcts (SBIs) in patients with asymptomatic carotid stenosis. We found that approximately 17% of these patients exhibit SBIs, which may be associated with more advanced carotid stenosis and a higher-risk cardiovascular profile. The observed associations suggest that certain hemodynamic factors and arterial wall characteristics could contribute to the development of these infarcts.

## Data Availability

The raw data supporting the conclusions of this article will be made available by the authors without undue reservation.

## TAXINOMISIS silent ischemia working group

From *Unit of Medical Technology and Intelligent Information Systems, Department of Materials Science and Engineering – Institute of Molecular Biology and Biotechnology, Department of Biomedical Research-FORTH, University Campus of Ioannina, Ioannina, Greece:* Dimitrios I. Fotiadis, Panagiotis K. Siogkas, Vassiliki Potsika, Vassilis Tsakanikas, and Vassiliki Kigka. From *Department of Vascular and Endovascular Surgery, School of Medicine & Klinikum rechts der Isar, Technical University of Munich, Munich, Germany:* Michael Kallmayer, Christoph Knappich, Christoph Schäffer, Stefan Saicic, Lan Zang, Felix Kirchhoff, Marie Kristin Fritsche. From *Fundacio per la Recerca Clinic Barcelona-IDIBAPS (FRCB-IDIBAPS):* Víctor Obach, Vicenç Riambau, Gaspar Mestres, Xavier Yugueros, Laula Llull. From *Vascular and Endovascular Surgery Clinic Ospedale Policlinico San Martino, Genova, Italy, Department of Integrated Surgical and Diagnostic Sciences (DISC), University of Genova, Genova, Italy:* Domenico Palombo, Giovanni Spinella, Bianca Pane, Giovanni Pratesi, Cristina Pallini, Jorge Miguel Mena Vera. From *Functional and Molecular Neuroimaging Unit, IRCCS Istituto delle Scienze Neurologiche di Bologna, Bologna, Italy:* Raffaele Lodi, Caterina Tonon, Luigi Cirillo, David Neil Manners. From the *Department of Medical and Surgical Sciences (DIMEC), University of Bologna, Bologna, Italy:* Alessandro Carrozzi. From *Vall d’Hebron Research Institute, Vall d’Hebron Barcelona Hospital Campus, Barcelona, Spain and Neuroradiology Unit, Radiology Department, Hospital del Mar, Barcelona, Spain:* Laura Ludovica Gramegna. From *Vascular Surgery, IRCCS Policlinico Sant’Orsola-Malpighi, Bologna, Italy:* Gianluca Faggioli, Rodolfo Pini, Mauro Gargiulo, Andrea Varcica. From *Department of Vascular Surgery, University Medical Center Utrecht, Utrecht, The Netherlands:* Ger Jan de Borst, Dominique P. V. de Kleijn, Simone Donners. From *Medical Faculty University of Belgrade (UBEO):* Davidovic Lazar, Igor Koncar, Miroslav Markovic, Andreja Dimic, Nikola Ilic, Marko Dragas, Ana Starcevic. From *Clinic for Vascular and Endovascular Surgery, University Serbian Clinical Center, Belgrade, Serbia:* Perica Mutavdzic, Marija Jovanovic, Ognjen Kostic, David Matejevic, Aleksandra Vujcic, Petar Zlatanovic, Branko Gakovic, Dragan Vasic. From *Vascular Unit, First Department of Propaedeutic Surgery, National and Kapodistrian University of Athens, Athens, Greece:* Franziska Sigala, George Galyfos, Georgios Karagiannis, Konstantinos Filis.
