# Supplementary material for: Silent brain ischemia within the TAXINOMISIS framework: association with clinical and advanced ultrasound metrics
Source: Front Neurol. 2024 Oct 11;15:1424362. doi: 10.3389/fneur.2024.1424362 (PMC11520771; doi:10.3389/fneur.2024.1424362)
Supplement: Supplementary file 1 [file Data_Sheet_1.docx]

**SUPPLEMENTARY MATERIALS**

**Part 1 - Baseline Study, centers analysis**

**UBEO**

In the UBEO cohort, of the 114 patients who underwent baseline MRIs, 5 (4.4%) were symptomatic presenting a total of 6 infarcts ipsilaterally relative to the symptomatic carotid artery. Among them, 3/5 (60%) patients had cortical infarcts, 1/5 (20%) had a lacunar infarct, and 1/5 (20%) had both a cortical and a lacunar infarct.

The remaining 109 (95.6%) patients were asymptomatic. Among these individuals, there were 14 (12.8%) subjects displaying 17 infarcts ipsilaterally to the included carotid artery. Among them, 8/14 (57.1%) had cortical infarcts, 1/14 (7.1 %) had lacunar infarcts, 2/14 (14.3%) had subcortical infarcts, and 3/14 (21.4%) had both cortical and subcortical infarcts.

Seven patients (6.1%) were previously operated on the contralateral carotid. All of them had asymptomatic stenosis of the included carotid.

**TUM**

In the TUM cohort, of the 49 patients who underwent baseline MRIs, 3 (6.1%) were symptomatic presenting a total of 3 infarcts ipsilaterally relative to the symptomatic carotid artery. Among them, 1/3 (33.3%) had cortical infarcts, none had lacunar infarcts, and 2/3 (66.7%) had subcortical infarcts. [*for use: Please notice there was one subcortical contralaterally].*

The remaining 46 (93.9%) patients were asymptomatic. Among these individuals, 11 (23.9%) displayed 12 infarcts ipsilaterally to the included carotid artery. Among them, 7/11 (63.6%) had cortical infarcts, 1/11 (9.1%) had lacunar infarcts, 2/11 (18.2%) had subcortical infarcts, and 1/11 (9.1%) had both a cortical and a lacunar infarct.

One patient (2.0%) was previously operated on the contralateral carotid. He/She had asymptomatic stenosis of the included carotid.

**USMI**

In the USMI cohort, of the 30 patients who underwent baseline MRIs, all (100%) were asymptomatic. Four patients (%) had 4 infarcts ipsilaterally to the included carotid artery. Among them, 1/4 (25%) had cortical infarcts, 1/4 (25%) had lacunar infarcts, and 2/4 (50%) subcortical infarcts.

**FCRB**

In the FCRB cohort, of the 18 patients who underwent baseline MRIs, all (100%) were asymptomatic.  Three patients had 3 infarcts ipsilaterally to the included carotid artery. Among them, 2/3 (66.7%) had cortical infarcts, and 1/3 (33.3%) had lacunar infarcts.

*[for use only: 3 patients had 4 contralateral infarcts. In dept, two patients had a contralateral cortical infarct, one both a contralateral cortical and subcortical].*

**Table 1**. Subjects characteristics in the Class 0 (Absence of Brain lesion) and Class 1 (Presence of Brain lesion) for cortical, lacunar, small subcortical, regarding the utilized dataset.

|  | **Class 0**  **(Absence of Cortical)**  **N=162** | **Class 1**  **(Presence of Cortical)**  **N=22** | **P value** | **Class 0**  **(Absence of Subcortical)**  **N=163** | **Class 1**  **(Presence of Subcortical)**  **N=9** | **P value** | **Class 0 (Absence of Lacunar)**  **N=163** | **Class 1 (Presence of Lacunar)**  **N=5** | **P value** |
| --- | --- | --- | --- | --- | --- | --- | --- | --- | --- |
| ICA stenosis | 63.78±13.936 | 67.71±12.697 | 0.916 | 63.78±13.936 | 65.5±15.14 | 0.655 | 63.78±13.94 | 67.25±9.84 | 0.544 |
| PSV | 174.65±97.84 | 211.2±107.35 | 0.200 | 174.65±97.84 | 206.11±94.61 | 0.909 | 174.65±97.84 | 158.5±126.41 | 0.561 |
| PSV ICA / PSV CCA | 3.83±6.319 | 4.31±2.22 | 0.930 | 3.83±6.32 | 3.68±2.33 | 0.863 | 3.83±6.32 | 3.25±2.89 | 0.915 |
| St Mary's ratio | 13.04±8.01 | 17.49±13.72 | **0.003** | 13.04±8.01 | 15.9±13.07 | 0.201 | 13.04±8.01 | 12.83±15.65 | 0.052 |
| PICA/PCCA | 0.92±0.17 | 0.97± 0.03 | 0.431 | 0.92± 0.17 | 0.98±0.01 | 0.518 | 0.92±0.17 | 0.98±0.0005 | 0.537 |
| PECA/PCCA | 0.89±0.31 | 0.94 ±0.06 | 0.514 | 0.89 ±0.31 | 0.95±0.02 | 0.535 | 0.89±0.31 | 0.97±0.02 | 0.621 |
| Vessel average TAWSS | 11.83±17.49 | 6.71±4.17 | 0.457 | 11.83± 17.49 | 6.67±2.33 | 0.541 | 11.83±17.49 | 7.74±0.15 | 0.521 |
| Area of low TAWSS (m^2^) | 0.0002±0.0002 | 0.0004±0.0003 | 0.141 | 0.0002± 0.0002 | 0.0006±0.0007 | **<0.001** | 0.0002±0.0002 | 0.0002±0.0000003 | 0.083 |
| Area of lowTAWSS/Total vessel area (%) | 23 ±17.1 | 42.07±27.04 | 0.209 | 23± 17.1 | 27.72±13.92 | 0.678 | 23±17.1 | 18.78±3.2 | 0.188 |
| Area of high OSI/Total vessel area (%) | 25.93±19.84 | 26.44±9.95 | 0.463 | 25.93±19.84 | 26.88±10.52 | 0.587 | 25.93±19.84 | 29.09±4.19 | 0.406 |
| Height | 168.08±16.67 | 172.35±9.59 | 0.722 | 168.08±16.67 | 169.13±10.03 | 0.894 | 168.08±16.67 | 174.6±8.14 | 0.736 |
| Weight | 75.75±11.8 | 78±8.92 | 0.170 | 75.75±11.80 | 77±9.46 | 0.385 | 75.75±11.8 | 89±10.2 | 0.529 |
| BMI | 26.39±3.35 | 26.32±2.17 | 0.27 | 26.39±3.35 | 27.12±4 | 0.528 | 26.39±3.35 | 29.42±4.86 | 0.234 |
| SBP | 130.97±16.48 | 131±17.023 | 0.778 | 130.97±16.48 | 135.56±14.41 | 0.932 | 130.97±16.48 | 140±14.71 | 0.987 |
| DBP | 80.06±8.49 | 77.55±11.4 | 0.052 | 80.06±8.49 | 80.22±8.21 | 0.838 | 80.06±8.49 | 85. ±4.3 | 0.273 |
| Puls rate | 70.67±7.52 | 69.71±7.37 | 0.848 | 70.67±7.52 | 70.57±4.12 | 0.297 | 70.67±7.518 | 72±2.65 | 0.279 |
| Age | 69.8±7.72 | 70.23±6.06 | 0.193 | 69.80±7.72 | 71±8 | 0.945 | 69.80±7.72 | 74.4±8.99 | 0.459 |
| Hb | 11.93±2.89 | 13.03±2.56 | 0.043 | 11.93±2.89 | 12.66±2.99 | 0.869 | 11.93±2.89 | 11.76±1.672 | 0.054 |
| Hct | 40.37±4.58 | 40.9±4.25 | 0.924 | 40.37±4.58 | 43.34±5.42 | 0.951 | 40.37±4.58 | 42.8±7.82 | 0.145 |
| Creatinine | 84.69±23.67 | 84.88±23.88 | 0.871 | 84.69±23.67 | 92.81±27.91 | 0.42 | 84.69±23.67 | 130.46±55.95 | **0.003** |
| Cholesterol | 4.67±1.1 | 4.34±1.17 | 0.702 | 4.67±1.1 | 4.34±1.33 | 0.437 | 4.67±1.1 | 4.53±1.29 | 0.801 |
| LDL | 2.6±1.02 | 2.3±0.77 | 0.311 | 2.6±1.02 | 2.21±1.14 | 0.916 | 2.6±1.03 | 2.64±0.72 | 0.379 |
| HDL | 1.52±0.56 | 1.4525±0.66 | 0.271 | 1.52±0.56 | 1.48±0.47 | 0.847 | 1.52±0.56 | 1.01±0.31 | 0.338 |
| Triglycerides | 1.48±0.78 | 1.77±1.07 | 0.165 | 1.48±0.78 | 1.78±0.95 | 0.415 | 1.48±0.78 | 2.48±1.77 | **<0.001** |
| Glucose | 6.42±2.28 | 5.88±2.36 | 0.769 | 6.42±2.28 | 6.72±2.59 | 0.603 | 6.42±2.28 | 6.46±2.85 | 0.596 |
| CRP | 3.47±3.9 | 2.22±1.85 | 0.202 | 3.47±3.9 | 3.41±3.82 | 0.74 | - | - | - |
| HbA1c | 6.02±0.84 | 6.24±1.37 | 0.155 | 6.02±0.8428 | 6.64±1.9 | **0.004** | - | - | - |

**Table 2**. Odds Ratio analysis for the association of CVD related risk factors and the presence of Brain lesions (cortical, small subcortical and lacunar).

| **Features** | **Presence of Cortical Infarct** | **Odds Ration -CI** | **P value** | **Presence of Subcortical Infarct** | **Odds Ration -CI** | **P value** | **Presence of Lacunar Infarct** | **Odds Ration -CI** | **P value** |
| --- | --- | --- | --- | --- | --- | --- | --- | --- | --- |
| Sex (male), n(%) | 17 (15%) | 0.428 (0.15-1.217) | 0.103 | 6 (5.8%) | 0.735 (0.177-3.042) | 0.67 | 5 (4.9%) | 0.951 (0.91-0.994) | 0.068 |
| Sex (female), n(%) | 5 (7%) |  |  | 3 (4.3%) |  |  | 0 (0 %) |  |  |
| Alcohol abuse, (no), n(%) | 19 (11.9%) | 1.391 (0.371-5.224) | 0.623 | 6 (4.1%) | 4.438 (1.01119.477-) | **0.033** | 5 (3.4%) | 0.966 (0.937-0.996) | 0.454 |
| Alcohol abuse (yes), n(%) | 3 (15.8%) |  |  | 3 (15.8%) |  |  | 0 (0%) |  |  |
| Diabetes(no), n(%) | 15 (11.5%) | 1.177 (0.451-3.073) | 0.739 | 7 (5.7%) | 0.705 (0.141-3.519) | 0.669 | 4 (3.3%) | 0.617 (0.067-5.666) | 0.667 |
| Diabetes (yes), n(%) | 7 (13.2%) |  |  | 2 (4.1%) |  |  | 1 (2.1%) |  |  |
| Hypertension (no), n(%) | 5 (17.9%) | 0.567 (0.19-1.686) | 0.302 | 1 (4.2%) | 1.324 (0.158-11.085) | 0.795 | 0 (0%) | 1.036 (1.004-1.069) | 0.364 |
| Hypertension (yes), n(%) | 17 (11%) |  |  | 8 (5.4 %) |  |  | 5 (3.5%) |  |  |
| Coronary Disease (no), n (%) | 15 (10.9%) | 1.406 (0.508-3.892) | 0.511 | 4 (3.1%) | 3.514 (0.836-14.772) | 0.07 | 3 (2.4%) | 2.343 (0.377-14.578) | 0.348 |
| Coronary Disease (yes), n(%) | 6 (14.6%) |  |  | 4 (10.3%) |  |  | 2 (5.4%) |  |  |
| Previous MI (no), n(%) | 18 (11.2%) | 1.833(0.476-7.056) | 0.372 | 7 (4.7%) | 1.571 (0.179-13.775) | 0.681 | 5 (3.4%) | 0.966 (0.938-0.996) | 0.501 |
| Previous MI (yes), n(%) | 3 (18.8%) |  |  | 1 (7.1%) |  |  | 0 (0%) |  |  |
| Previous Cabg/pci (no), n(%) | 16 (10.5%) | 2.253 (0.74-6.857) | 0.144 | 4 (2.8%) | 7.211 (1.664-31.254) | **0.003** | 4 (2.8%) | 1.803 (0.191-16.987) | 0.602 |
| Previous Cabg/pci (yes), n(%) | 5 (20.8%) |  |  | 4 (17.4%) |  |  | 1 (5%) |  |  |
| Atherosclerosis of aortiliac/femoropoplitealcrural (no), n(%) | 17 (13.5%) | 0.611 (0.194-1.921) | 0.395 | 6 (5.2%) | 0.845 (0.164-4.351-) | 0.84 | 5 (4.4%) | 0.956 (0.919-0.994) | 0.163 |
| Atherosclerosis of aortiliac/femoropoplitealcrural (yes), n(%) | 4 (8.7%) |  |  | 2 (4.4%) |  |  | 0 (0%) |  |  |
| Aortic aneurysm (no), n(%) | 19 (11.5%) | 2.561(0.482-12.609) | 0.254 | 7 (4.6%) | 3.476 (0.367- 32.939) | 0.249 | 5 (3.3%) | (-) |  |
| Aortic aneurysm (yes), n(%) | 2 (25%) |  |  | 1 (14.3%) |  |  | 0 (0%) |  |  |
| Alpha-Blockers (no), n(%) | 20 (11.3 %) | 5.233 (0.824-33.24) | 0.052 | 9 (5.4%) | 0.946 (0.912-0.981) | 0.9 | 5 (3.1%) | 0.969 (0943-0.996) | 0.721 |
| Alpha-Blockers (yes), n(%) | 2 (40%) |  |  | 0 (0%) |  |  | 0 (0%) |  |  |
| Beta-Blockers (no), n(%) | 9 (10.7%) | 1.275 (0.516-3.15) | 0.599 | 4 (5.1%) | 1.09 (0.282-4.208) | 0.9 | 2 (2.6%) | 1.308 (0.213-8.04-) | 0.771 |
| Beta-Blockers (yes), n(%) | 13 (13.3%) |  |  | 5 (5.5%) |  |  | 3 (3.4%) |  |  |
| Diuretics, (no), n(%) | 17 (13.4%) | 0.647 (0.226-1.852) | 0.414 | 6 (5.1%) | 1.11 (0.267-4.618) | 0.886 | 4 (3.5%) | 0.555 (0.06-5.093) | 0.598 |
| Diuretics, (yes), n(%) | 5 (9.1%) |  |  | 3 (5.7%) |  |  | 1 (2%) |  |  |
| Cachannel blockers (no), n(%) | 17 (12.4%) | 0.882 (0.306-2.545) | 0.817 | 7 (5.5%) | 0.864 (0.172-4.331) | 0.859 | 4 (3.2%) | 0.756 (0.082-6.965) | 0.805 |
| Cachannel blockers (yes), n(%) | 5 (11.1%) |  |  | 2 (4.8%) |  |  | 1 (2.4%) |  |  |
| ACE inhibitors (no), n(%) | 17 (17.2%) | 0.317 (0.112-0.902) | 0.025 | 4 (4.7%) | 1.331 (0.345-5.14) | 0.677 | 4 (4.7%) | 0.266 (0.029-2.435) | 0.210 |
| ACE inhibitors (yes), n(%) | 5 (6.2%) |  |  | 5 (6.1%) |  |  | 1 (1.3%) |  |  |
| Angiotensin receptors antagonist (no), n(%) | 16 (11%) | 1.625 (0.587-4.501) | 0.347 | 8 (5.8%) | 0.546 (0.066-4.531) | 0.57 | 3 (2.2%) | 2.911 (0.466-18.196) | 0.233 |
| Angiotensin receptors antagonist (yes), n(%) | 6 (16.7%) |  |  | 1 (3.2%) |  |  | 2 (6.3%) |  |  |
| Other antihypertensive drugs (no), n(%) | 20 (12%) | 1.131(0.238-5.383) | 0.877 | 9 (5.7%) | 0.943 (0.907-0.98) | 0.375 | 5 (3.3%) | 0.967 (0.94-0.996) | 0.508 |
| Other antihypertensive drugs (yes), n(%) | 2 (13.3%) |  |  | 0 (0%) |  |  | 0 (0%) |  |  |
| Coronary drugs (no), n(%) | 21 (12.1%) | 1.041 (0.122-8.885) | 0.971 | 8 (4.9%) | 2.75 (0.301-25.132) | 0.351 | 4 (2.5%) | 5.5 (0.541-55.884) | 0.108 |
| Coronary drugs (yes), n(%) | 1 (12.5%) |  |  | 1 (12.5%) |  |  | 1 (12.5%) |  |  |
| Anticoagulants (no), n(%) | 20 (11.2%) | 7.9 (1.054-59.221) | 0.019 | 8 (4.8%) | 6.583 (0.614-70.566) | 0.075 | 5 (3.1%) | 0.969 (0.943-0.996) | 0.758 |
| Anticoagulants (yes), n(%) | 2 (50%) |  |  | 1 (25%) |  |  | 0 (0%) |  |  |
| Acetylsalicylic acid (no), n(%) | 2 (10.5%) | 1.189 (0.255-5.535) | 0.825 | 0 (0%) | 1.063(1.021-1.106) | 0.289 | 2 (10%) | 0.189 (0.03-1.207) | 0.051 |
| Acetylsalicylic acid (yes), n(%) | 20 (12.3%) |  |  | 9 (5.9%) |  |  | 3 (2.1%) |  |  |
| Clopidogrel (no), n(%) | 18 (11.5%) | 1.471 (0.454-4.771) | 0.518 | 9 (6.1%) | 0.939 (0.901-0.978) | 0.235 | 4 (2.8%) | 1.58 (0.169-14.792) | 0.686 |
| Clopidogrel (yes), n(%) | 4 (16%) |  |  | 0 (0%) |  |  | 1 (4.3%) |  |  |
| Statin (no), n(%) | 4 (21.1%) | 0.466 (0.139 -1.556) | 0.205 | 1 (6.3%) | 0.822 (0.096-7.026) | 0.858 | 1 (6.3%) | 0.411 (0.043-3.918) | 0.425 |
| Statin(yes), n(%) | 18 (11%) |  |  | 8 (5.2%) |  |  | 4 (2.7%) |  |  |
| Lipidlowering drugs(LLD) (no), n(%) | 20 (12.3%) | 0.835 (0.179-3.889) | 0.818 | 5 (3.4%) | 5.047 (1.107-23.01) | 0.022 | 4 (2.7%) | 2.103 (0.222-19.917) | 0.508 |
| Lipidlowering drugs(LLD) (yes), n(%) | 2 (10.5%) |  |  | 3 (15%) |  |  | 1 (5.6%) |  |  |
| Contralateral (no), n(%) | 17 (10.7%) | 2.088 (0.694 6.283-) | 0.182 | 3 (2.1%) | 14.3 (3.312-61.745) | **<0.001** | 3 (2.1%) | 4.767 (0.75-30.296) | 0.07 |
| Contralateral (yes), n(%) | 5 (20%) |  |  | 6 (23.1%) |  |  | 2 (9.1%) |  |  |
| Ever operated on contralateral carotid (no), n(%) | 19 (13%) | 0.477 (0.105-2.169) | 0.329 | 4 (3.1%) | 3.402 (0.721-16.057) | 0.103 | 5 (3.8%) | 0.962 (0.93-0.995) | 0.295 |
